# Hypovitaminosis D among Adult Patients Visiting for General Health Check-up in a Tertiary Care Center: A Descriptive Cross-sectional Study

**DOI:** 10.31729/jnma.8612

**Published:** 2024-06-30

**Authors:** Sanjeeb Tiwari, Saroj GC, Vijay Shrees, Sujata Maharjan, Aakash Sherpali, Khagendra Bhandari, Suman Oli

**Affiliations:** 1 Department of General Practice, Tribhuvan University Teaching Hospital, Maharajgunj, Kathmandu, Nepal; 2 Maharajgunj Medical Campus, Maharajgunj, Kathmandu, Nepal; 3 Sindhuli Hospital, Kamalamai, Sindhuli, Nepal.

**Keywords:** *adults*, *prevalence*, *tertiary care center*, *vitamin D deficiency*

## Abstract

**Introduction:**

Vitamin D deficiency presents a notable public health concern, with reported prevalence rising in hospital and community settings. It's linked to various chronic health issues and most often remains undiagnosed in developing nations. This study aimed to determine the prevalence of hypovitaminosis D among adults attending general health check-ups at a tertiary care hospital.

**Methods:**

This descriptive cross-sectional study was conducted among adult patients visiting for general health checkups in a tertiary care centre. The patients' data from 16 April 2023 to 24 November 2023 was retrieved from the hospital record. Serum 25(OH)D was measured by using the chemiluminescence micro particles immunoassay technique and classified as deficient, insufficient, and sufficient with values <20 ng/ml, 20-29 ng/ml, and 30-100 ng/ml, respectively. A convenience sampling method was used. The point estimate was calculated at a 95% Confidence Interval.

**Results:**

Out of 357 adult patients, 291 (81.51%; 95% CI: 77.49%-85.54%) Confidence Interval) had hypovitaminosis D. Among them 124 (42.61%) were categorised as vitamin D insufficient and 167 (57.39%) as deficient. The mean age of patients was 43.25±12.99 years, with 205 (70.45%) female and 86 (29.55%) male. A total of 169 (58.08%) individuals were classified as obese. Dyslipidemia was observed in 249 (85.57%) patients, with 94 (32.30%) exhibiting hypercholesterolemia.

**Conclusions:**

The prevalence of hypovitaminosis D was higher than other studies done in similar settings. This higher prevalence necessitates public awareness of vitamin D's importance, urging proactive screening and management by physicians and implementation of cost-effective guidelines by policymakers.

## INTRODUCTION

Vitamin D deficiency represents a significant global public health challenge necessitating prioritisation for its prevention due to its persistent high prevalence.^1^ It is an independent risk factor for various health complications including cardiovascular disease, cancer, autoimmune disorders, infectious diseases, and other chronic conditions contributing to increased all-cause mortality in the general population.^2^

The existing studies in Nepal have primarily focused on sick patients within hospital or community settings with largely unknown general health statuses. Studies in Nepal have revealed a prevalence of vitamin D deficiency ranging from 55.9% to 73.6%.^3-5^ The change in lifestyle, dietary patterns, and environmental factors may have exacerbated the issue, emphasising the need for a strategic plan of action.^2^

The objective of this study was to find out the prevalence of hypovitaminosis D among adult patients visiting for general health checkups in a tertiary care hospital.

## METHODS

A descriptive cross-sectional study was conducted among patients visiting for general health checkups in the Department of General Practice, Tribhuvan University Teaching Hospital (TUTH), Maharajgunj, Kathmandu, Nepal after obtaining ethical approval from the Institutional Review Committee of Institute of Medicine, Tribhuvan University (Reference number: 378 (6-11)E2 080/081). The patients' data from 16 April 2023 to 24 November 2023 was retrieved from the hospital record. These data were collected from 05 January 2024 to 25 February 2024. Adult patients aged 18 years and older, undergoing general health checkups, and those having vitamin D level analysis reports during the study period were included in the study. Those patients undergoing blood tests for reasons outside routine health screening (sick visits or acute illness), suffering from skin, liver, and kidney diseases, and those on vitamin D supplementation were excluded from the study. A convenience sampling method was used. The sample size was calculated using Cochran's sample size formula (one sample situation for binary data): n = Z^2^pq/e^2^, at 95% level of significance and allowable error (e) at 5%.

The tabulated value of Z at 95% level of significance was 1.96, Z^2^ = (1.96)^2^ = 3.84; prevalence of hypovitaminosis D(p) = 72.87%^5^, q = (100-p) = 27.13; e^2^ = 25. The calculated minimum required sample size was 304. However, we have included 357 patients in our study.

The predesigned proforma containing clinical, sociodemographic, and laboratory parameters (including lipid profile) was used to retrieve data from the hospital record.

Serum 25(OH)D was analyzed in the Department of Biochemistry of TUTH. The collected venous blood samples were centrifuged to separate the serum. Serum 25(OH)D was measured by using the chemiluminescence micro particles immunoassay technique (Abbott architect ci4100) and internal quality control was maintained as per the guides from the manufacturer.

Serum 25(OH)D was classified as deficient, insufficient, and sufficient with values <20 ng/ml, 20-29 ng/ml, and 30-100 ng/ml, respectively.^6^ Further, the severity of vitamin D deficiency is categorized as mild if 25-hydroxyvitamin D levels are less than 20 ng/mL, moderate if less than 10 ng/mL, and severe if less than 5 ng/mL.^6^

Smoking status was classified as a current smoker or never smoker. Alcohol intake was divided into alcoholic beverages and did not drink. Body mass index (BMI) was calculated by dividing weight by height squared (kg/meter2) and classified as underweight (<18.5 kg/m^2^), normal weight (18.5-22.9 kg/m^2^), overweight (≥23.0-24.99 kg/m^2^), and obese (≥25 kg/m^2^).^7^ Dyslipidemia was classified based on the criteria outlined in the third report of the National Cholesterol Education Program Adult Treatment Panel (NCEP ATP III), with the following threshold values: hypercholesterolemia, defined as a serum total cholesterol (TC) level of ≥200 mg/dL; hypertriglyceridemia, defined as a serum triglyceride (TG) level of ≥150 mg/dL; low high-density lipoprotein cholesterol (HDL-C) level, defined as ≤40 mg/dL for both men and women; and high low-density lipoprotein cholesterol (LDL-C) level, defined as ≥100 mg/dL.^8^

Data was coded and entered in Microsoft Excel 2019 and analyzed by using IBM SPSS Statistics version 16.0. A point estimate at a 95% CI was calculated. Descriptive statistics was evaluated as frequency and percentage for binary data; and mean and standard deviation for normally distributed continuous data.

## RESULTS

**Figure 1:**
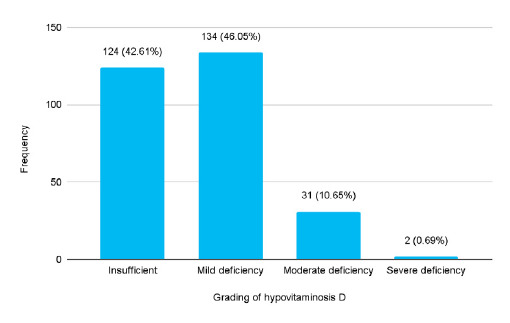
The severity distribution of patients with hypovitaminosis D (n= 291).

Among 357 adult patients, the prevalence of hypovitaminosis D was 291 (81.51%) (95% CI: 77.4985.54%). Among them, 124 (42.61%) were vitamin D insufficient and 167 (57.39%) were vitamin D deficient (Figure 1).

There were 86 (29.55%) male and 205 (70.45%) female with hypovitaminosis D. The mean age of patients was 43.25±12.99 (18-86) years. Regarding age distribution, the largest proportion fell within the 40-49 age group (26.12%), followed by the 30-39 age group (25.09%). In terms of ethnicity, the Brahman group constituted the largest segment (37.80%), followed by Janajati (29.21%) and Chhetri (23.71%) (Table 1).

**Table 1:** Socio-demographic parameters of patients with hypovitaminosis D (n = 291).

Variables	n (%)
**Gender**	
Male	86 (29.55)
Female	205 (70.45)
**Age group (years)**	
<20	3 (1.03)
20-29	43 (14.78)
30-39	73 (25.09)
40-49	76 (26.12)
50-59	66 (22.68)
60-69	21 (7.22)
70-79	7 (2.41)
≥80	2 (0.69)
**Ethnicity**	
Brahman	110 (37.80)
Janajati	85 (29.21)
Chhetri	69 (23.71)
Occupational caste (Dalit)	14 (4.81)
Newar	13 (4.47)

Among the observed lifestyle factors, 25 patients (8.59%) reported smoking, while 47 (16.15%) reported alcohol consumption. Among comorbidities, a total of 35 individuals (12.03%) had hypertension, 13 (4.47%) had diabetes mellitus, and 16 (5.50%) were diagnosed with a thyroid disorder. Within the group of patients with thyroid disorders, 15 (5.15%) were diagnosed with hypothyroidism. A total of 169 (58.08%) individuals were classified as obese, while dyslipidemia was observed in 249 (85.57%) patients, with 94 (32.30%) of them presenting with hypercholesterolemia (Table 2).

**Table 2:** Clinical and laboratory parameters of patients with hypovitaminosis D (n = 291).

Variables	n (%)
Smoking	25 (8.59)
Alcohol	47 (16.15)
Hypertension	35 (12.03)
Diabetes mellitus	13 (4.47)
Thyroid disorder	16 (5.50)
**BMI**	
Obese	169 (58.08)
Overweight	53 (18.21)
Normal weight	58 (19.93)
Underweight	11 (3.78)
**Serum lipid profile**	
Dyslipidemia	249 (85.57)
Hypercholesterolemia	94 (32.30)
Low HDL-C	132 (45.36)
High LDL-C	189 (64.95)
Hypertriglyceridemia	109 (37.46)

## DISCUSSION

The prevalence of hypovitaminosis D among adult patients in our study was 81.51%. Comparable studies determined a prevalence of 73.6% among outpatients at Kathmandu Medical College and Teaching Hospital, Sinamangal, Kathmandu, and 72.87% among patients at the Universal College of Medical Sciences and Teaching Hospital, Rupandehi.^4,5^ Similarly, a study from Lalitpur found that 90.20% of adult outpatients with multiple complaints like backache, muscle pain, leg pain, etc were vitamin D deficient.^9^ A community- based study from eastern Nepal observed a prevalence of 55.9%.^3^ Community-based studies often include a broader, more diverse population, including healthy individuals, which might lower the overall prevalence of deficiency observed as compared to our study.

The studies collectively demonstrate a high prevalence of vitamin D deficiency across various populations. In the United States, the overall prevalence is 41.65%, with particularly high rates among Blacks and Hispanics, and significant associations with race, education level, obesity, health status, and dietary habits.^10^ An urban Beijing study reported an 87.1% prevalence of vitamin D deficiency, emphasizing the urgent need for targeted interventions, especially among young and elderly females during winter and spring.^11^ Research in Jeddah, Saudi Arabia, found that 87.8% of men had vitamin D deficiency, predominantly affecting older, obese, sedentary, and uneducated individuals.^12^ Similarly, studies conducted in North India, and France showed a prevalence of vitamin D deficiency of 88.9%^13^, and 92.3%^14^ respectively. Our study showed that 42.61% were vitamin D insufficient and 57.39% were deficient. Likewise, the study from central Nepal-Chitwan reported a 70.7% hypovitaminosis D prevalence among which deficiency was 34.8% and insufficiency in 35.9%.^15^

Differences in mineral metabolism across various racial groups can influence the effects of calcium and vitamin D supplementation, particularly among nonWhite populations.^16^ The main source of vitamin D is its production in the skin through exposure to sunlight, yet factors like high latitudes and lifestyle choices can restrict this process. Variables such as air pollution, residential latitude, and skin pigmentation can compromise the intensity of UV rays, affecting vitamin D synthesis.^17^ Variations in prevalence can arise from differences in study settings, inclusion criteria for age groups, and the demographic diversity of the populations under investigation. Despite advancements in vitamin D testing, significant variability in assay results due to genetic, environmental, and methodological factors necessitates international guidelines and potentially individualized centile curves to accurately assess vitamin D status, as fixed limits may not be appropriate. This might also have contributed to the variability of the proportion of vitamin D deficiency status in the studies.^18^

In our study, the mean age of patients was 43.25±12.99 years, while studies in Chitwan and Rupandehi reported mean ages of 47.2±16.9 and 45.35±15.96 years, respectively.^5,15^ In our study, Brahman and Chhetri ethnicities comprised approximately 61.51%, whereas other studies indicated a proportion of 35.8%.^3^ Among patients with hypovitaminosis D in our study, 70.45% were female and 29.55% were male, while other studies reported 29.20% male and 70.80% female.^5^ Similarly, the overall prevalence of hypovitaminosis D was higher in females 72.4% than in 64.2% of males.^15^ A systematic review and meta-analysis estimated the pooled burden in South Asia to be 68%, with Pakistan having the highest burden followed by Bangladesh, India, Nepal, and Sri Lanka, respectively. This analysis also found mean vitamin D levels ranging from 4.7 to 32 ng/ml, with higher prevalence in females than males and significant heterogeneity among populations.^19^ In contrast, a meta-analysis from African populations showed a lower prevalence of 17-31% when compared to similar vitamin D deficiency cut-off (below 30 nmol/L), considerably less than in South Asian regions. They found a pooled prevalence of 34.18% for concentrations below 50 nmol/L and 58.54% for concentrations below 75 nmol/L. Serum 25(OH)D levels were lower in urban areas than rural areas, among women versus men, and in newborns compared to their mothers.^20^

In our study, 169 individuals (58.08%) were classified as obese, while dyslipidemia was observed in 249 (85.57%) of the adult patients. Vitamin D deficiency is associated with increased obesity and body fat, and while its impact on the severity of obesity remains unclear, it is consistently linked to atherosclerotic cardiovascular disease and unfavourable lipid profiles, which are significant contributors to global mortality rates. The association between vitamin D deficiency and lipid profiles in overweight and obese adults was investigated through a meta-analysis of 21 articles involving 7952 participants. Results indicated that individuals with vitamin D deficiency exhibited significantly higher levels of triglycerides, total cholesterol, and low-density lipoprotein cholesterol, while also showing reduced levels of high-density lipoprotein cholesterol compared to controls. These findings suggest that vitamin D deficiency is associated with impaired lipid profiles among overweight and obese adults, highlighting the potential role of vitamin D status in lipid metabolism and cardiovascular health in this population.^21^

Convenience sampling was employed, potentially introducing selection bias due to differences in health behaviours, awareness, and socioeconomic status between participants seeking general health checkups and the general population, thereby limiting the generalizability of findings. Due to financial constraints, not all patients undergoing general health check-ups were assessed for vitamin D levels, thus restricting the analysis to the available data only. Furthermore, the study duration did not capture all seasonal variations in vitamin D levels, potentially overlooking specific temporal patterns. Single-point measurements of vitamin D levels may not adequately reflect long-term status, thus potentially missing variations over time. The study did not extend beyond the available data to explore interrelationships between conditions.

The higher prevalence emphasizes the need for public awareness regarding the importance of vitamin D for health and the adverse consequences of its deficiency. Further, longitudinal studies are recommended to identify factors associated with hypovitaminosis D and preventive strategies relevant to our study population.

## CONCLUSIONS

The prevalence of hypovitaminosis D among adult patients visiting for general health check-ups in our tertiary care centre was higher as compared to other studies done in similar settings.
